# Perfluorooctanesulfonate Mediates Renal Tubular Cell Apoptosis through PPARgamma Inactivation

**DOI:** 10.1371/journal.pone.0155190

**Published:** 2016-05-12

**Authors:** Li-Li Wen, Chien-Yu Lin, Hsiu-Chu Chou, Chih-Cheng Chang, Hau-Yin Lo, Shu-Hui Juan

**Affiliations:** 1 Graduate Institute of Medical Sciences, Taipei Medical University, Taipei, Taiwan; 2 Department of Clinical Laboratory, En Chu Kong Hospital, New Taipei City, Taiwan; 3 Department of Medical Laboratory Science and Biotechnology, Yuanpei University, Hsinchu, Taiwan; 4 Department of Internal Medicine, En Chu Kong Hospital, New Taipei City, Taiwan; 5 School of Medicine, Fu Jen Catholic University, New Taipei City, Taiwan; 6 Department of Anatomy and Cell Biology, School of Medicine, College of Medicine, Taipei Medical University, Taipei, Taiwan; 7 Department of Physiology, School of Medicine, College of Medicine, Taipei Medical University, Taipei, Taiwan; University of Hawaii Cancer Center, UNITED STATES

## Abstract

Perfluorinated chemicals (PFCs) are ubiquitously distributed in the environments including stainless pan-coating, raincoat, fire extinguisher, and semiconductor products. The PPAR family has been shown to contribute to the toxic effects of PFCs in thymus, immune and excretory systems. Herein, we demonstrated that perfluorooctanesulfonate (PFOS) caused cell apoptosis through increasing ratio of Bcl-xS/xL, cytosolic cytochrome C, and caspase 3 activation in renal tubular cells (RTCs). In addition, PFOS increased transcription of inflammatory cytokines (i.e., TNFα, ICAM1, and MCP1) by NFκB activation. Conversely, PFOS reduced the mRNA levels of antioxidative enzymes, such as glutathione peroxidase, catalase, and superoxide dismutase, as a result of reduced PPARγ transactivational activity by using reporter and chromatin immuoprecipitation (ChIP) assays. PFOS reduced the protein interaction between PPARγ and PPARγ coactivator-1 alpha (PGC1α) by PPARγ deacetylation through Sirt1 upregulation, of which the binding of PPARγ and PGC1α to a peroxisome proliferator response element (PPRE) in the promoter regions of these antioxidative enzymes was alleviated in the ChIP assay. Furthermore, Sirt1 also deacetylated p53 and then increased the binding of p53 to Bax, resulting in increased cytosolic cytochrome C. The effect of PPARγ inactivation by PFOS was validated using the PPARγ antagonist GW9662, whereas the adverse effects of PFOS were prevented by PPARγ overexpression and activators, rosiglitozone and L-carnitine, in RTCs. The *in vitro* finding of protective effect of L-carnitine was substantiated *in vivo* using Balb/c mice model subjected to PFOS challenge. Altogether, we provide *in vivo* and *in vitro* evidence for the protective mechanism of L-carnitine in eliminating PFOS-mediated renal injury, at least partially, through PPARγ activation.

## Introduction

Perfluorinated chemicals (PFCs) are materials with special properties that have many critical manufacturing and industrial applications. Despite the production and use of PFCs for the past 60 years, concerns regarding the environmental hazards of these compounds arose only recently, and literature regarding human and wildlife exposure is increasing [1]. PFCs comprise perfluorohexane sulfonic acid (PFHxS, a 6-carbon PFC), perfluorooctane sulfonic acid (PFOS, an 8-carbon PFC), perfluorooctanoic acid (PFOA, an 8-carbon PFC), and perfluorononanoic acid (PFNA, a 9-carbon PFC). PFOS is the dominant PFC, followed by PFOA and PFHxS [2]. Serum levels for fluorochemical plant workers are in the 1–2 mg/L range. The serum levels in the general public are 17–53 μg/L for PFOS and 3–17 μg/L for PFOA [3, 4]. The half-life of serum elimination of PFCs in humans appears to be years. The longer the carbon chain, the longer the PFCs persist in the body. For example, half-life of perfluorobutane sulfonate (a 4-carbon PFC) is, on average, in slightly more than 1 month in humans [5], whereas the half-lives of PFOA and PFOS are in 3.5 and 4.8 years, respectively.

PFCs have been associated with numerous health effects in animal and human studies. A previous study showed that serum PFCs have been detected in greater than 98% of the US population, which is associated with several risk factors for diseases, including increased total and low-density lipoprotein cholesterol [6, 7], increased uric acid levels [6, 8], increased risk of diabetes and metabolic syndrome [9], and tumorigenicity [10, 11] in human epidemiological studies. Higher concentrations of PFOS and PFOA were observed in the kidneys [12, 13] because they are the primary route for PFCs excretion [14]. In addition, rats exposed to PFOA and PFOS cause renal hypertrophy and histopathologic changes, suggesting the involvement of soft tissue proliferation in the renal interstitium and renal microvascular disease [2]. Results of *in vitro* studies have also indicated that PFCs are correlated with alterations in endothelial cell permeability [15, 16], which are believed to be a central mechanism underlying ischemic renal failure in rat models [17]. Furthermore, an epidemiological study has shown that serum PFOS and PFOS were positively correlated with chronic kidney disease [18]. However, the results were not entirely consistent [19], and the causal relationship and mechanism underlying the effects of PFOS in renal tubular cells (RTCs) remain unknown.

The possible mechanisms underlying PFC-mediated toxicity in wildlife and humans are contradictory and remain inconclusive. Several studies have shown that PFOS and PFOA can activate PPARα in humans and mice [20]. As compared to PFOA, PFOS was shown to be less effective in activating PPARα, and both PFOS and PFOA were shown to have no significant activating effect on PPARγ [21]. Midgett et al. also showed that PFOS at environmentally related concentrations does not significantly increase the induction of PPAR-α, γ, or β genes [22]. In addition, the hypothesis that other mechanisms independent of PPARα modulate the effects induced by PFCs was supported by microarray analysis and reporter gene assays, which revealed that in addition to PPARα, PFCs activate other transcription factors including PPARβ, PPARγ, CAR, and PXR [23]. Therefore, compelling evidence exists, suggesting that the PFCs-mediated toxicities are not mediated by one single receptor. Likewise, a recent study on PPARα-knockout mice demonstrated that the developmental toxicity of PFOS (including reduction in body weight and an increased prenatal mortality) was not dependent on the PPARα pathway [24]. The mechanisms through which PFOS produces developmental toxicity remain unclear. Furthermore, one possible toxicity mechanism underlying PFOS exposure has been reported to alter mitochondrial biogenetics [25, 26], although few studies on the relation between PFOS toxicity and mitochondria have been conducted.

L-carnitine (L-trimethyl-3-hydroxy-ammoniabutanoate), a quaternary ammonium compound, is synthesized from lysine and methionine amino acids [27]. In lipid catabolism, it is required to transport fatty acids from the cytosol into the mitochondria and sold as the nutritional supplement. L-carnitine has been shown to induce anti-oxidative molecules (i.e., endothelial nitric oxide synthase, heme oxygenase-1 (HO-1), and superoxide dismutase (SOD) [28] and protects phospholipid membranes from lipid peroxidation and cardiomyocytes and endothelial cells from oxidative stress [29]. Furthermore, our group previously showed that L-carnitine can prevent gentamicin-induced apoptosis in RTCs by PPARα activation through a prostaglandin (PG) I2-depedent pathway [30]. Recently, L-carnitine was also shown to reduce hypertension-associated renal fibrosis in a PPARγ-dependent manner [31].

Silent information regulator T1 (Sirt1), a NAD-dependent deacetylase sirtuin-1, deacetylates a wide range of substrates including PPARγ, p53, NFκB, FOXO transcription factors, and PGC-1α, with roles in cellular processes ranging from energy metabolism to cell survival [32]. However, SIRT1 also has been shown to facilitate the mitochondrial dependent apoptotic response by controlling p53 subcellular localization in mouse embryonic stem cells through blocking cytoplasmic p53 nuclear translocation [33]. Increasing evidence shows that human Sirt1 is highly expressed in cancer cell lines and that a strong cytosolic component exists in the Sirt1 expression pattern [34]. Furthermore, the aberrant cytoplasmic localization and protein stability of Sirt1 were elucidated to be regulated by PI3K/IGF-1R signaling in human cancer cells [35]. Hence, Sirt1 is implicated in a wide range of human diseases and is a prominent therapeutic target. Mammalian sirtuins (e.g., SIRT1) exhibiting HDAC activity inhibit 3T3-L1 adipogenesis and promote fat mobilization in white adipocytes by repressing PPARγ through their interaction with it [36]. Okazaki et al. added to the complexity of the overall control of fatty acid metabolism by showing that the transcription of the Sirt1 gene is itself controlled by fatty acid metabolism, which is also regulated by PPARβ through Sp1 [37]. Likewise, animal studies have shown that starvation increases lipolysis-derived free fatty acids and activates PPARβ, resulting in the upregulation of Sirt1 expression. To our knowledge, the effect of the interplay between Sirt1 and the PPAR family is mainly limited to adipocytes; however, their impact on renal function requires further investigation.

Herein, the adverse effects and mechanism of action of PFOS on RTCs and kidney function were analyzed. We revealed the mechanisms underlying the detrimental effects of PFOS on RTC apoptosis and proposed therapeutic prevention by using L-carnitine through a Sirt1/PPARγ-dependent mechanism.

## Materials and Methods

### Cell culture and reagents

We used the rat renal proximal tubular epithelial cell line, NRK-52E, for the *in vitro* RTC model in our study. NRK-52E epithelial cell lines are composed of differentiated, anchorage-dependent, non-tumorigenic cells that undergo density-dependent inhibition of proliferation [38]. The widely used NRK-52E rat kidney cell lines have been characterized with the morphological and kinetic properties of kidney tubule epithelial cells [39]. The NRK-52E cells were purchased from the Bioresource Collection and Research Center (Hsinchu, Taiwan) and were cultured in Dulbecco’s modified Eagle medium (DMEM) supplemented with 10% fetal bovine serum (FBS) and an antibiotic and antifungal solution. The NRK-52E cell monolayers were grown until they reached confluence. The DMEM, FBS, and other tissue culture reagents were obtained from Life Technologies (Gaithersburg, MD, USA). Sirtinol was purchased from Santa Cruz Biotechnology (Dallas, Texas, USA), and GW9662 was acquired from Enzo Life Science Inc. (Farmingdale, New York, USA). All the other chemicals were of the reagent grade and were obtained from Sigma-Aldrich (St. Louis, MO, USA).

### Analysis of gene transcription by using reverse transcription-PCR

The method of obtaining the total RNA for the RT-PCR analysis was as described previously [40], with minor modifications. The sequences of the primer pairs for the amplification of each gene were listed in Table 1. Five micrograms of the total RNA from the RTC extracts were used. The expression of the housekeeping gene GAPDH was analyzed and used to demonstrate the presence of the same amount of total cDNA in each RNA sample.

**Table 1:** Pairs of primers for genes of the interest in RT-PCR analysis.

Gene name	Forward primer	Reverse primer	Product size (bps)
**TNFα**	TGCCTCAGCCTCTTCTCATT	CCCATTTGGGAACTTCTCCT	108
**ICAM-1**	AGGTATCCATCCATCCCACA	GCCACAGTTCTCAAAGCACA	209
**MCP-1**	ATGCAGTTAATGCCCCACTC	TTCCTTATTGGGGTCAGCAC	167
**Gpx-1**	AGAAGTGCGAGGTGAATGGT	CGGGGACCAAATGATGTACT	127
**SOD-1**	GGAGAGCATTCCATCATTGG	CAATCACACCACAAGCCAAG	127
**catalase**	ACATGGTCTGGGACTTCTGG	AAGGTGTGTGAGCCATAGCC	121
**GAPDH**	AACTTTGGCATTGTGGAAGG	TGTTCCTACCCCCAATGTGT	223

### Preparation of cell fractions (nuclear and cytosolic) and western blot analysis

RTCs were harvested in 10-cm^2^ dishes after the indicated treatment. The cells were partitioned into cytosolic and nuclear fractions by using NE-PER^TM^ nuclear extraction reagents (Pierce, Rockford, IL, USA) with the addition of protease inhibitors, according to the manufacturer’s instructions. Antibodies against PPARγ (SC-7273), Bcl-xL/xS (SC-1041), NFAT3 (SC-13036), NFκB-p65 (SC-372), PGC1α (SC-13067), Sirt1 (SC-15404), pan-acetylated (SC-8649) and Lamin A/C (SC-6215) (1:500 Santa Cruz Biotechnology, Santa Cruz, CA, USA), caspase 3 (#13909; 1:500, Cayman Chemical, Ann Arbor, MI, USA), GAPDH (#LF-PA0018; 1:2000, Ab Frontier, Seoul, Korea), and β-actin (#MABT523; 1:500, Millipore, Burlington, MA, USA) were included in the assay. The cell lysate (50 μg) was electrophoresed on an 8% sodium dodecylsulfate-polyacrylamide gel and then transblotted onto a Hybond-P membrane (GE Healthcare, Hong Kong SAR). The subsequent procedures were as described previously [41]. Western blot bands were quantified by using the Scion Image Software (Scion, Frederick, MD).

### Cell proliferation and determination of apoptosis in RTCs and renal tissue sections

The effect of PFOS in RTC proliferation was assessed by using Cellometer Mini cell counter (Lawrence, MA, USA). The number of cells in each of triplicate wells was counted after 24 and 40 h of PFOS treatment. To determine the effect of PFOS in RTC apoptosis, RTCs were cultured on poly-L-lysine-coated 0.17-mm coverslips, and the cell numbers were counted at 50%–80% confluence, followed by the indicated treatments. The cells were then fixed in 4% formaldehyde for 15 min. The cells were permeabilized using 0.2% Triton and 0.1% Tween-20 in blocking buffer (3% bovine serum albumin in phosphate-buffered saline [PBS]) for 2 h. Four coverslips in each experimental group were examined. Apoptosis in RTCs challenged with PFOS for 24 h was identified by performing a terminal deoxynucleotidyl transferase dUTP nick end-labelling (TUNEL) assay by using an *in situ* Cell Death Detection kit (Roche, Mannheim, Germany) according to the manufacturer’s instructions. Fluorescence was visualized using a CCD camera (DP72, Olympus, Melville, NY, USA) attached to a microscope system (BX51, Olympus) at 100× magnification.

### Coimmunoprecipitation (CoIP)

The Sirt1/PPARγ/PGC1α complexes were immunoprecipitated from 200 μg of protein by using anti-PPARγ antibody (2 μg) and protein A plus G agarose beads (20 μg), followed by western blot analysis for the protein levels of Sirt1, acetylated-PPARγ and PGC1α. The precipitates were washed five times with lysis buffer and once with PBS. The pellet was then resuspended in sample buffer (50 mM Tris [pH 6.8], 100 mM bromophenol blue, and 10% glycerol) and incubated at 90°C for 10 min before electrophoresis to release the proteins from the beads.

### Luciferase activity assay of the peroxisome proliferator response element reporter (PPRE) and chromatinimmunoprecipitation (ChIP) assay

The pBV-luc plasmid containing the prototypic peroxisome proliferator response element (PPRE) (5′-AGGTCAAAGGTCA-3′) from the acyl-CoA oxidase gene promoter was a gift from Dr. Vogelstein (Johns Hopkins University, Baltimore, MD, USA) [42]. The pSV-Sport plasmid containing full length cDNA of murine PPARγ1 was a gift from Dr. Lin Teng-Nan (Academia Sinica, Institute of Biomedical Sciences, Taipei, Taiwan), which was purchased from Addgene (Cambridge, MA, USA). The method for the reporter activity assay was as described previously [43]. A chromatinimmunoprecipation (ChIP) assay was performed according to the instructions of Upstate Biotechnology (Lake Placid, NY, USA) with minor modifications. In brief, 6 × 10^5^ cells that were cultured in 100-mm dishes with the indicated treatments were harvested. The resulting supernatant was subjected to overnight CoIP by using an anti-PGC1α or anti-PPARγ antibody. The DNA filtrates were amplified by carrying out a PCR with primers flanking the promoters of Gpx-1, SOD-1, and catalase containing the putative PPREs. Sequences of the primer pairs for the amplification of each gene were as follows: 5′-CCCCCACTCAACTGGACTAA-3′ and 5′-GAACTCTGCACCAAAGCACA-3′ for the Gpx-1 (200 bp); 5′-GTCGCAACTGAGGTTGGATT-3′ and 5′-GCCAGCTACCAACCAAGAGA-3′ for the SOD-1 promoter (135 bp); and 5′-GGTCTCAAAGGAGCCATGAA-3′ and 5′-GGGATCTGTGAAGGGTCTGA-3′ for the catalase promoter (189 bp). The PCR products were electrophoresed on a 2% agarose gel, and the products of the expected sizes were visualized and quantified using an image analysis system.

### Animals and treatments

All animal study procedures were conducted in accordance with the Taipei Medical University animal care and use rules (licenses No. LAC-2014-0233) and Institutional Animal Care and Use Committee or Panel (IACUC/IACUP). Eight-week-old male Balb/c mice weighing 20–25 g were obtained from the Research Animal Center at National Taiwan University (Taipei, Taiwan). The animals were housed in a central facility, subjected to a 12-h light–dark cycle, and were provided regular rat chow and tap water. PFOS (potassium salt; >91% pure) was dissolved in 0.5% Tween-20 in PBS, and all dosing solutions were freshly prepared daily. Selection of the dose and period was based on the study of PFOS [44] and our previous study of L-carnitine [45]. Forty-eight Balb/c mice were separated into control (0.5% Tween-20 in PBS), PFOS (1, 10 mg/kg), L-carnitine (50 mg/kg), and L-carnitine (50 mg/kg) + PFOS (1, 10 mg/kg) groups. To evaluate the harmful effect of PFOS in murine kidneys, the mice were alternatively IP-injected with L-carnitine or PFOS every other day and 3 times/week for 2 weeks (n = 8). At the end of the treatment period, animals were anaesthetized intramuscularly with a combination of ketamine (8 mg/100 g body weight), xylazine (2 mg/100 g) and atropine (0.16 mg/100 g). The murine kidneys were obtained for the analysis of cell apoptosis and western blot analysis of the Bcl-xL, Bcl-xS, and caspase-3 levels. Blood samples were collected to measure the serum levels of creatinine and urea nitrogen by using Fuji Dri-Chem slides (Fujifilm, Tokyo, Japan). The kidneys were harvested by performing a laparotomy, and tissue samples of the renal cortex were snap frozen in dry ice and subsequently stored at −80°C. The renal tissue samples were fixed in 10% formalin and embedded in paraffin. Serial 5-μm sections were prepared from the paraffin-embedded samples from the control and PFC-treated groups, and the sections were stained with hematoxylin and eosin (H&E) for histological analysis. Frozen sections (5 μm, n = 8) were also prepared for TUNEL assay.

### Statistical analysis

The data are expressed as the mean ± SEM, representing the results of at least three experiments. The significance of the difference between the control and each experimental test condition was analyzed by Student’s *t* test and between two experimental groups was estimated by one-way analysis of variance. *P* < 0.05 was considered statistically significant.

## Results

### Concentration-dependent effects of PFOS on apoptosis and inflammation and the prevention by L-carnitine in RTCs

**Fig 1:**
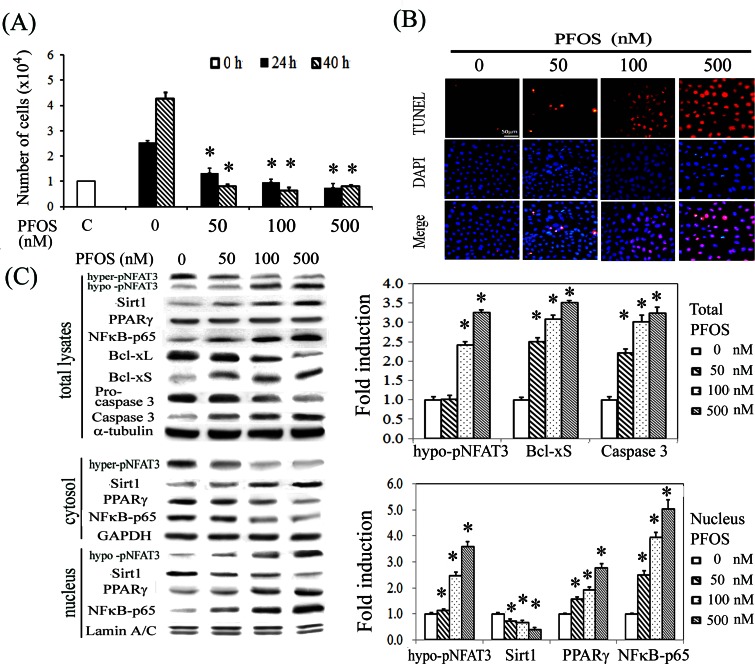
Prooxidative and inflammatory effects of PFOS in cell proliferation and apoptosis. (A) RTC proliferation was assessed after 24 and 40 h of PFOS treatment by using Cellometer Mini cell counter. (B) RTCs with 24 h of PFOS exposure were evaluated for apoptosis after staining for TUNEL assay. Red represents positive staining for apoptosis. Identical fields were stained with 4′,6-diamidino-2-phenylindole to reveal the positions of cell nuclei. Micrographs of representative fields were recorded, and the magnification was ×200. (C) Cells were treated with PFOS for 6 h for western blot analysis of NFAT3, Sirt1, PPARγ, and NFκB-p65 or for 18 h for Bcl-xL, Bcl-xS, and Caspase 3 analysis. Samples consisted of total cell lysates or cytosolic–nuclear fractions as indicated. The bar chart shows the normalized intensities of each protein band. Lamin A/C and GAPDH were used as internal controls for the nuclear and cytoplasmic fractions, respectively whereas alpha tubulin serves as loading control for whole cell lysates. Results are expressed as the mean ± SEM (n = 4). Data from a representative experiment are shown. Significant difference: *P < 0.05 vs. control.

We have performed pilot studies by using a wide-range concentration of PFOS (0–500 nM) and treatment endurance of 0–40 h, and chosen a lower concentration of 50 or 100 nM PFOS and indicated time points for the subsequent mechanistic study. We showed that RTCs challenged with PFOS reduced cell proliferation after 24 and 40 h of treatment in a concentration-dependent manner (Fig 1A), which was correlated with increased apoptosis by PFOS at 24 h, as observed in the TUNEL assay (Fig 1B) and significantly increased levels of proapoptotic molecules, Bcl-xS, and cleaved caspase 3, and reduced levels of the antiapoptotic protein Bcl-xL as observed in the western blot analysis (Fig 1C). The activation of NFAT3 through hypophosphorylation reflected an increased PFOS-induced oxidative stress, which was confirmed by an increase in NADPH oxidase activity as detected using a luminometer (data not shown). Notably, the levels of Sirt1 and NFκB-p65 in total cell lysates were increased in response to an increasing concentration of PFOS treatment (Fig 1C). Additionally, western blot analysis of the cell lysates after cytosolic–nuclear fractionation showed that PFOS significantly increased the nuclear translocation of NFAT3, PPARγ, and NFκB-p65 and reduced the nuclear level of Sirt1 in a concentration-dependent manner, indicating their potential involvement in PFOS-mediated RTC apoptosis.

**Fig 2:**
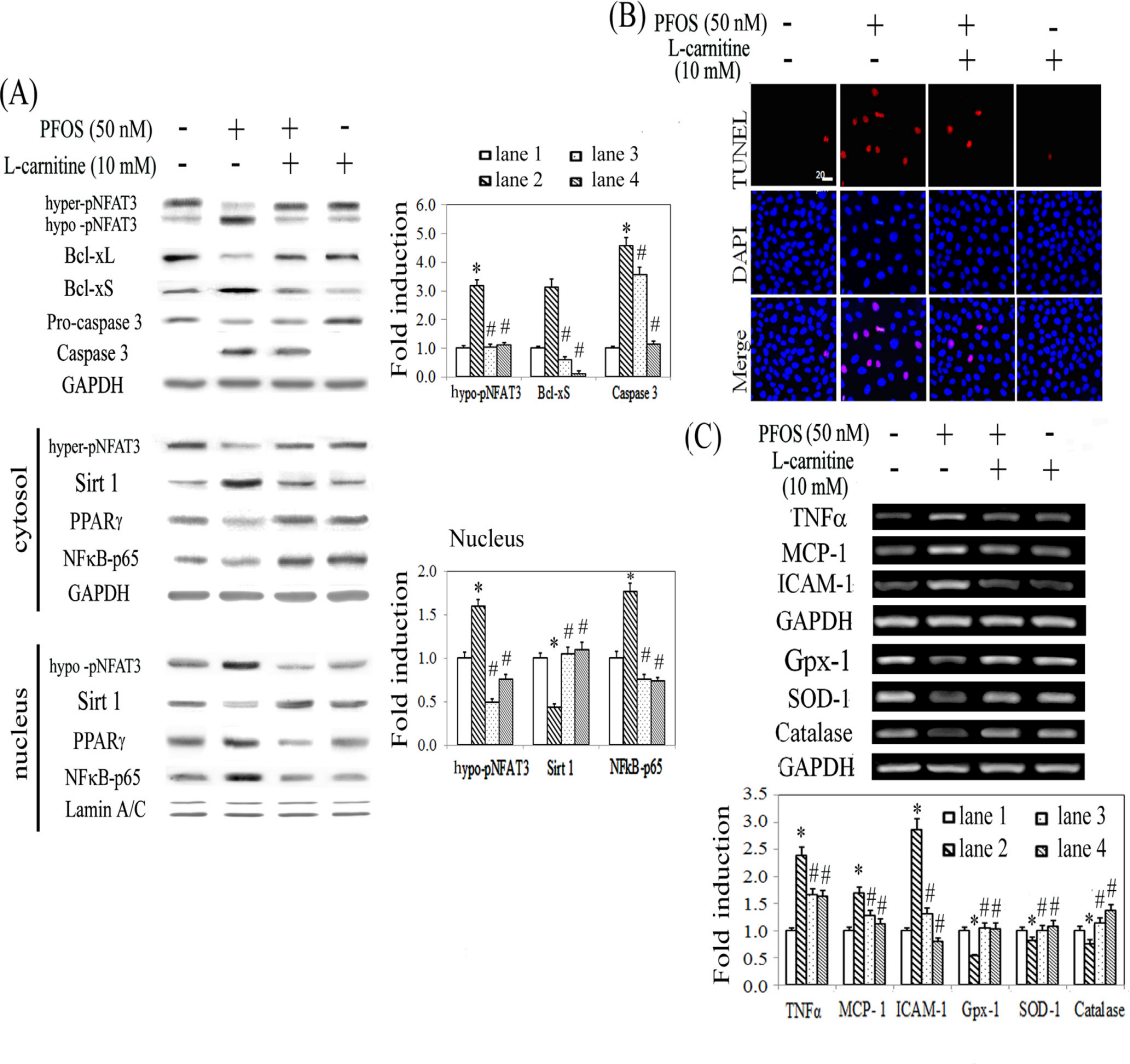
Protective effect of L-carnitine in PFOS-mediated RTC cytotoxicity through reduced Sirt1 cytosolic sequestration and increased expression of antioxidative enzymes. The methods used in (A) and (B) are similar to those in Fig 1, except that cells were pretreated with L-carnitine for 24 h, followed by the PFOS challenge. (C) L-carnitine-mediated alterations in inflammatory cytokines and antioxidative enzymes were analyzed using RT-PCR. RTCs were pretreated with L-carnitine for 24 h, followed by the PFOS challenge for 6 h to evaluate the effect of L-carnitine on PFOS-mediated mRNA transcription, including that of TNFα, MCP1, ICAM1, Gpx-1, SOD-1, and catalase. GAPDH was used as an internal control. The data are presented as the mean ± SEM of the results of four independent experiments (*P < 0.05 vs. control; ^#^*P* < 0.05 vs. PFOS-treated group).

L-carnitine possesses antioxidative and antiinflammatory effects and reduced hypertension-associated renal fibrosis in a PPARγ-dependent manner [31]. The efficacy of L-carnitine in PFOS-mediated renal injury was evaluated. Western blot analysis showed that L-carnitine reversed the detrimental effects of PFOS by suppressing the apoptotic signaling pathway (i.e., reduced the levels of Bcl-xS and cleaved form of caspase 3 and the nuclear translocation of NFκB-p65 and NFAT3) (Fig 2A). The anti-apoptotic effect of L-carnitine in PFOS-treated RTCs was shown in Fig 2B by using TUNEL assay. Furthermore, RT-PCR was used to examine the increased transactivation activity of NFκB-65 in cytokine production in PFOS-treated RTCs and the protective effect of L-carnitine. L-carnitine treatment alleviated the increase in inflammatory cytokines, including TNFα, MCP1, and ICAM1, and rescued the downregulation of antioxidative enzymes, including glutathione peroxidase (Gpx-1), catalase, and CuZn-SOD (SOD-1) induced by the PFOS challenge (Fig 2C).

### Alleviation of PPRE inactivation and apoptotic signaling by L-carnitine and rosiglitazone

**Fig 3:**
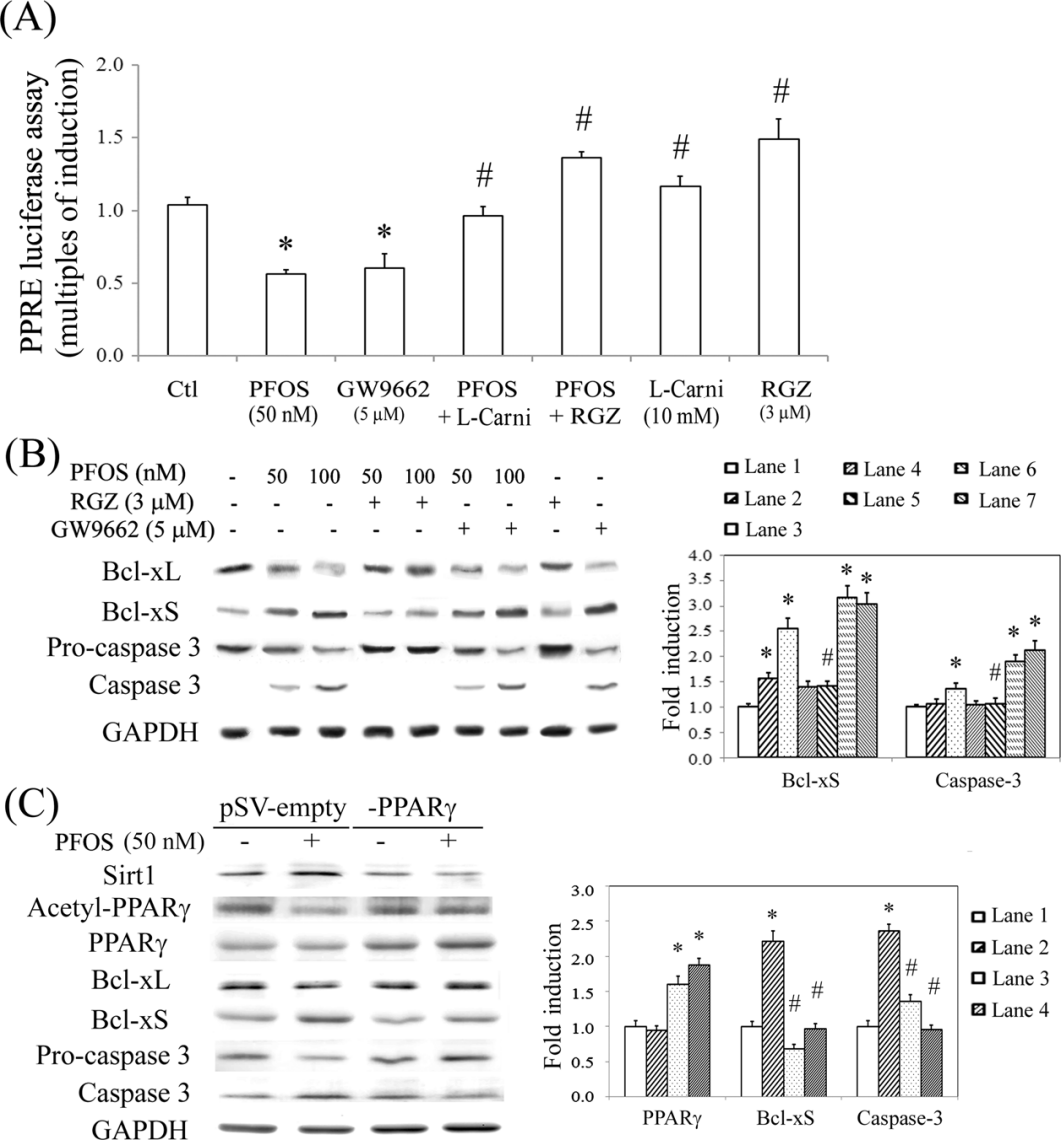
PFOS-mediated PPARγ inactivation by a luciferase reporter assay and rescue of RTC apoptosis by PPARγ agonists. (A) RTCs were transfected with the PPRE-luciferase vector overnight, followed by pretreatment of PPARγ agonist/antagonist for 1 h and then 2 h of PFOS challenge. Cells were harvested and analyzed for the PPRE-driven luciferase activity assay. The data were processed as described in the Methods section. (B) RTCs were pretreated with an agonist/antagonist of PPARγ for 1 h or (C) cells were transfected with pSV-empty or–PPARγ plasmid overnight, followed by 18 h of PFOS challenge for the analysis of Bcl-xL, Bcl-xS, and Caspase 3 protein levels. Cell lysates were analyzed using western blotting. Band intensities were normalized on the basis of the GAPDH band intensity by using densitometry. The bar chart in each panel shows the normalized intensities of each protein band. The data were derived from the results of three independent experiments and are presented as the mean ± SEM (*P < 0.05 vs. control; ^#^*P* < 0.05 vs. the corresponding concentration of the PFOS-treated group).

PFCs exert their action through a PPAR-dependent pathway. To verify this, we examined the effect of PFOS by performing a PPRE-driven luciferase assay in RTCs transfected with a PPRE-luciferase vector. Unexpectedly, PFOS reduced the PPRE-driven transactivational activity of luciferase in RTCs, which could be rescued using an additional treatment of L-carnitine and rosiglitazone (RGZ, a PPARγ agonist), but deteriorated by GW9662 (a PPARγ antagonist) (Fig 3A). To examine the protection by L-carnitine and RGZ, a western blot analysis was performed, which showed that L-carnitine (Fig 2A) and RGZ (Fig 3B) reverted the effect of PFOS by increasing the ratio of Bcl-xL to Bcl-xS, which consequently reduced the levels of the activated form of caspase 3. By contrast, the apoptotic effect of PFOS was exacerbated by GW9662. This suggested that the mechanism underlying the adverse effect of PFOS involved PPARγ inactivation, which was in turn prevented by PPARγ activators. Likewise, RTCs with PPARγ overexpression prevented cells from apoptosis with reduced levels of Bcl-xS and caspase 3 through inhibition of PPARγ deacetylation and Sirt1 induction (Fig 3C).

### Downregulation of antioxidative enzymes by eliminating the binding of PPARγ-PGC1α to PPRE through Sirt1-mediated PPARγ deacetylation

**Fig 4:**
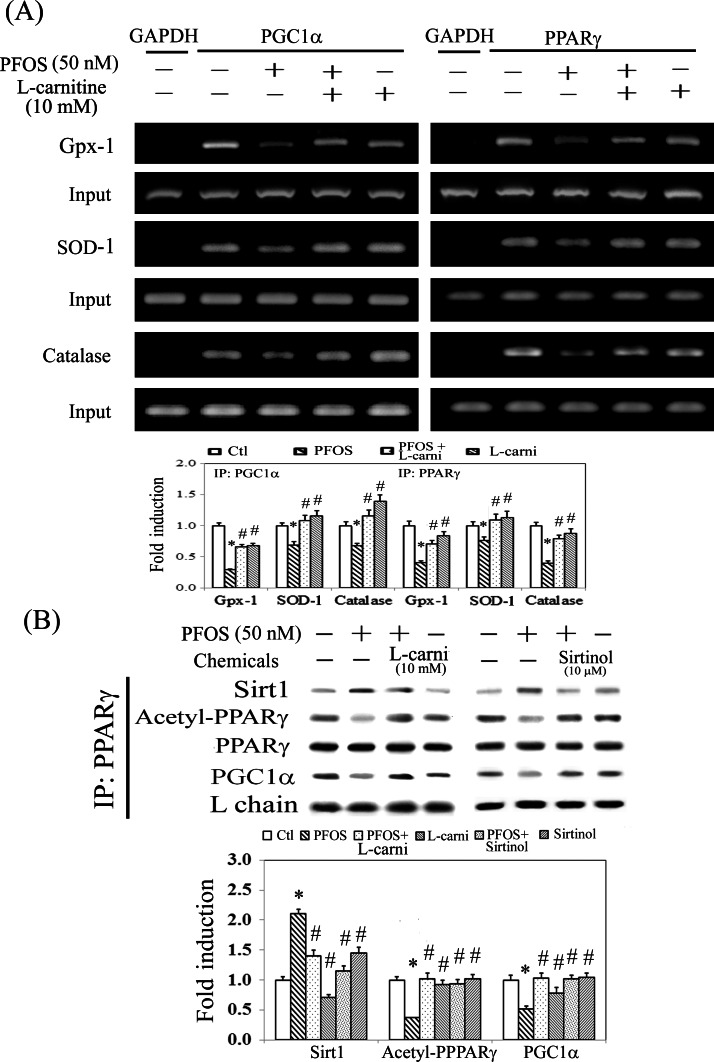
Reduced binding of PPARγ to the PPREs in the promoters of antioxidative enzymes and reduced protein interaction of PPARγ and PGC1α in PFOS-treated RTCs. (A) A ChIP assay was performed in cells that received L-carnitine (L-carni) pretreatment for 24 h, followed by the PFOS challenge for 2 h. The DNA–protein complex was immunoprecipitated with anti-PGC1α and PPARγ antibodies; subsequently, PCR amplification was employed to examine the association between PGC1α/PPARγ and the functional PPRE-binding sites in the promoters of Gpx-1, SOD-1, and catalase. An anti-GAPDH antibody was used as a negative control for the ChIP assays. (B) Cells were pretreated with L-carnitine for 24 h and Sirtinol for 1 h, followed by 6 h of PFOS treatment. The PPARγ-associated proteins were immunoprecipitated using an anti-PPARγ antibody, and the complexes were probed using antiacetylated, anti-Sirt1, and anti-PGC1α antibodies. The immunoprecipitation was normalized to the amount of PPARγ pulled down and an IgG light chain (L chain) was used as an input control. The data are representative of the results of three independent experiments, and the data are presented as the mean ± SEM (*P < 0.05 vs. control; ^#^*P* < 0.05 vs. PFOS treatment alone).

We speculated that the reduced levels of antioxidative enzymes (Gpx-1, SOD-1, and catalase) (Fig 2) resulted from PFOS-mediated PPARγ inactivation. Therefore, the promoters of these target genes were analyzed for the conserved PPRE by using MatInspector software. The oligonucleotides flanking the putative PPRE were synthesized and employed for PCR amplification in a ChIP assay. The results showed that the binding of PGC1α and PPARγ to the putative PPREs was significantly reduced in the PFOS-treated groups compared with the control groups after PCR amplification for the pull down of the protein–DNA complexes (Fig 4A). By contrast, additional L-carnitine can reverse this effect caused by PFOS. Furthermore, the results of the CoIP assay showed that Sirt1 is involved in PPARγ deacetylation and reduced the recruitment of PGClα (a coactivator of PPARγ) to PPARγ, resulting in PPARγ inactivation (Fig 4B). Likewise, this can be alleviated by using additional L-carnitine and Sirtinol (a Sirt1 inhibitor) treatment by suppressing Sirt1-mediated PPARγ deacetylation. This suggests that the essential role of Sirt1 in PPARγ deacetylation and inactivation resulted in the inhibitory transcriptional regulation of antioxidative enzymes in PFOS-treated RTCs.

### Effect of Sirt1 induction by PFOS in p53 deacetylation and increased protein interaction between p53 and Bax to increase cytosolic cytochrome C

**Fig 5:**
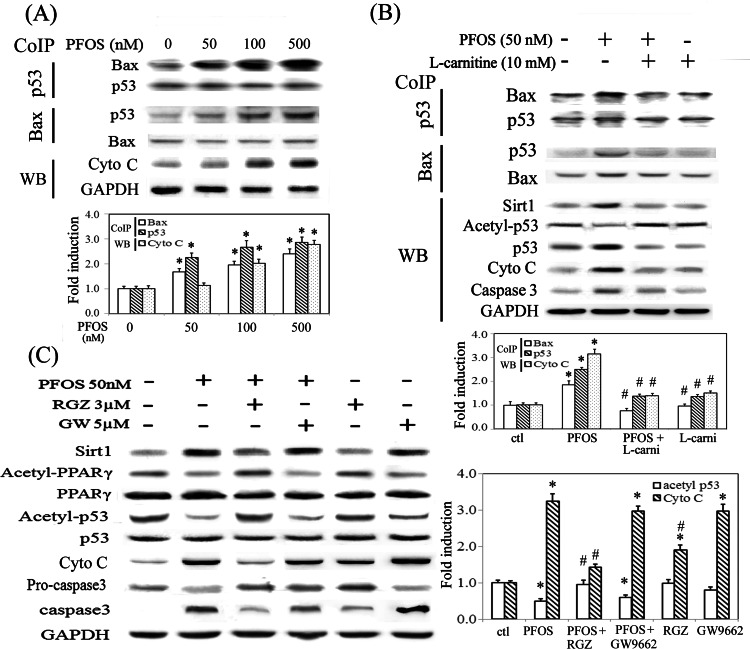
PPARγ activators abolished PFOS-mediated p53 deacetylation and increased cytosolic cytochrome C by reducing Sirt1 induction. (A) Cells were treated with the indicated concentrations of PFOS for 6 h to examine protein interaction between p53 and Bax in a Co-IP assay, and expression pattern of cytosolic cytochrome C in western blot analysis. (B) Cells were pretreated with L-carnitine for 24 h and challenged with PFOS for 6 h. The effect of L-carnitine in PFOS-treated RTCs was assessed for expression levels of Sirt1, acetyl-53, p53, cytochrome C and caspase 3 in western blot analysis, and protein interaction of p53 and Bax in a Co-IP assay. (C) Cells were pretreated with RGZ and GW9662 for 1 h and treated with PFOS for 6 h. Cell lysates were analyzed for the indicated molecules, as shown in western blots. The bar chart shows the normalized intensity of each protein band with respective p53 or Bax pulled down in Co-IP assays and with GAPDH in western blots. Results are expressed as the mean ± SEM (n = 4). Data from a representative experiment are shown. Significant difference: *P < 0.05 vs. control; ^#^*P* < 0.05 vs. PFOS treatment alone.

PFOS increased protein interaction between p53 and Bax in a concentration-dependent manner, accompanied with increased cytosolic level of cytochrome C and caspase 3 activation (Fig 5A). In PFOS-treated RTCs, p53 deacetylation by Sirt1 is associated with the increased protein interaction between p53 and Bax, and increased cytosolic levels of cytochrome C, whereas L-carnitine abolished the effect by reducing the level of Sirt1 (Fig 5B). Likewise, RGZ activated PPARγ to reduce Sirt1 induction, resulting in inhibition of p53 deacetylation, and reduced cytosolic cytochrome C and caspase 3 activation. On the contrary, GW9662 mimicked the effects of PFOS in RTCs (Fig 5C).

### Protective effect of L-carnitine in RTC apoptosis and renal morphology/function in mice challenged with PFOS

**Fig 6:**
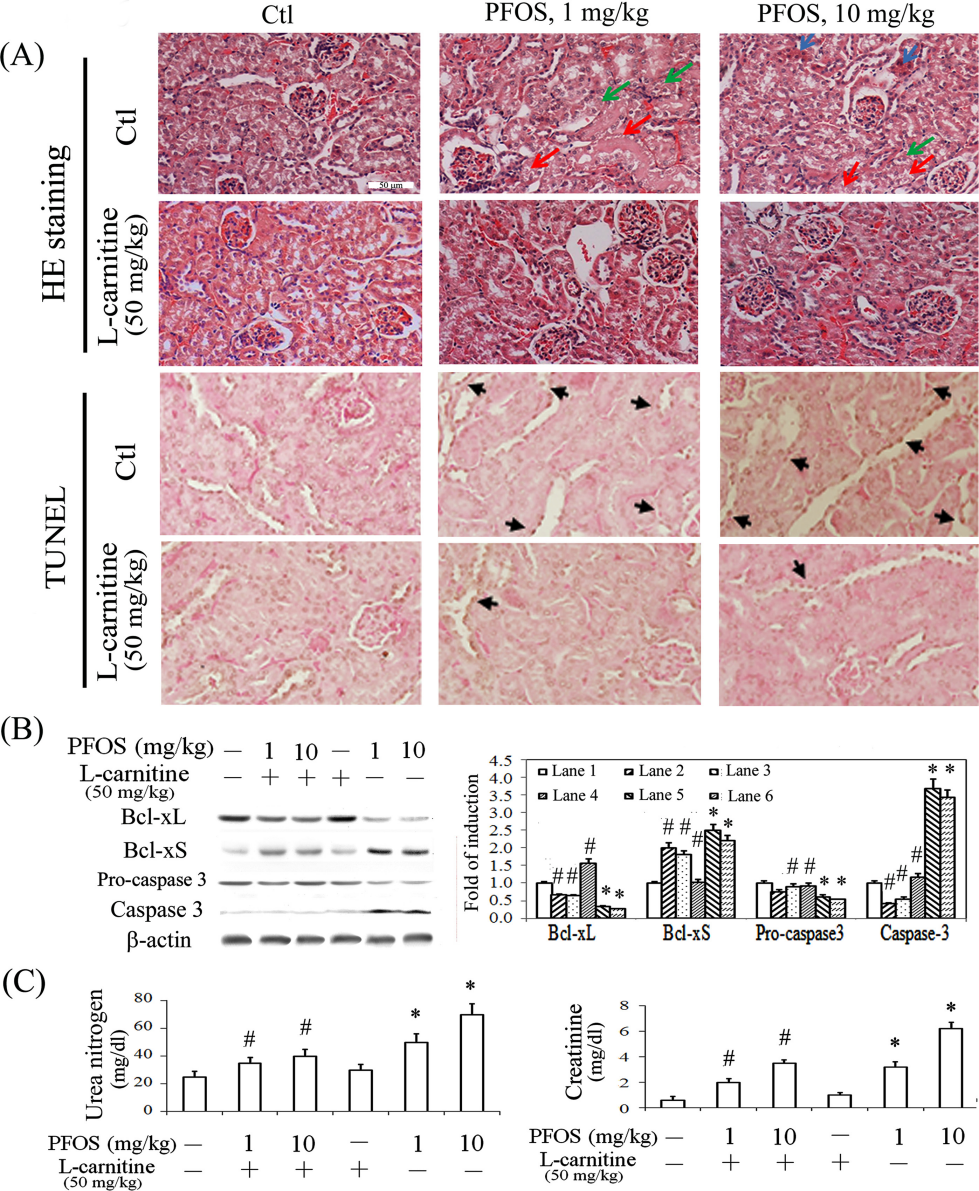
Prevention of PFOS-mediated renal apoptosis, morphological alterations, and renal function by using L-carnitine treatment *in vivo*. The protective effect of L-carnitine on PFOS-mediated changes in renal apoptosis and structure. The kidneys were dissected and sectioned for (A) histological examination and TUNEL assay. Representative photographs of H&E staining are shown in the top panel. Red arrow: cell death; blue arrow: granular cytoplasm of some proximal epithelial cells; and green arrow: drop out of some epithelial cells. Apoptotic cells in the kidneys of experimental animals were detected *in vivo* by using TUNEL staining. (Second panel) The TUNEL-labelled nuclei were visible as brownish spots (black arrow) in the cortical sections of untreated and treated mouse kidneys. White scale bar represents 50 μm. (B) Kidney extracts from mice with indicated treatments were subjected to western blot analysis for analyzing the levels of Bcl-xL/xS and procaspase 3/caspase 3, with β-actin as an internal control. (C) The serum levels of urea nitrogen and creatinine were measured after 2 weeks of treatment. The results are expressed as the mean ± SEM (n = 8; *P < 0.05 vs. control; ^#^*P* < 0.05 vs. PFOS-treated group).

We examined the in vivo protective effect of L-carnitine in PFOS-mediated renal apoptosis. Selection of the dose was based on the studies of PFOS [24, 44] to mimic the concentrations observed in occupational fluorochemical exposure and our previous study of L-carnitine to protect against renal injury [30, 45]. Mice challenged with PFOS demonstrated significantly cell death (red arrow), the loss of some epithelial cells (green arrow), the granular cytoplasm of some proximal renal tubular cells (blue arrow), reduced acidophilic features, a loss of their normal configurations, such as the microvilli (brush border) and an enlarged lumen of proximal or distal convoluted tubules (Fig 6A). By contrast, the mice with additional L-carnitine treatment can mitigate the PFOS-induced histological damage. Apoptotic cells in the kidneys of the experimental animals were detected *in vivo* by using TUNEL staining. Majority of the TUNEL-labelled nuclei were appeared in proximal renal tubular cells. The brownish-stained nuclei labelled by TUNEL (black arrow) were detected in the renal cortex of PFOS-treated mice; however, they scarcely occurred in those of the control and L-carnitine-treated mice. These results indicate that L-carnitine can significantly protect renal tubular cells from PFOS-mediated apoptosis. Western blot analysis showed that the reduced ratio of Bcl-xL to Bcl-xS and caspase 3 activation in renal tissues were reverted in mice following L-carnitine treatment (Fig 6B). Furthermore, the renal functional assays showed that mice with PFOS challenge exhibited increased serum urea and creatinine levels, indicative of the impairment on normal renal function (Fig 6C). However, this can be significantly prevented in mice by using additional L-carnitine treatment, suggesting the protection of L-carnitine against PFOS-mediated renal dysfunction.

## Discussion

The adverse effects of PFCs in humans and wildlife have attracted substantial attention. The underlying mechanism is generally believed to be PPAR-dependent. Herein, we demonstrated that PFOS caused RTC apoptosis by inhibiting expression of anti-oxidative enzymes, and increasing p53 and Bax interaction through a Sirt1/PPARγ-dependent mechanism. Additionally, PFOS increased NFAT3 activation through hypophosphorylation and induced cytokine production in RTCs.

The mechanism underlying PFOS toxicity remains controversial. Studies have shown that the detrimental effect of PFCs occurs through PPAR activation. However, by contrast, mice exposed to PFOS exhibited the upregulation of PPARγ and IL-1β in the thymus and spleen, resulting in the atrophy of immune organs [46]. IL-1β, a downstream target of NFκB, has been shown to be inhibited by PPARγ activation in monocytes and macrophages through the inhibition of proinflammatory transcription factors [47, 48]. Therefore, the increased IL-1β expression might indicate the inactivation of PPARγ, although PFOS upregulates PPARγ. Moreover, PPARα-knockout mice showed a reduction in body weight and an increased prenatal mortality independent of PPARα in response to the PFOS challenge [24]. Similarly, we showed that PFOS reduced PPRE-driven luciferase reporter activity, which was mimicked by GW9662 (a PPARγ antagonist); however, RGZ (a PPARγ agonist) activated the reporter (Fig 3A). Moreover, GW9662 mimicked the effect of PFOS in the cell apoptosis/inflammatory response, whereas RGZ and L-carnitine prevented the detrimental effect of PFOS in RTCs. The novelty of our study is to demonstrate the inhibitory effect of PFOS in PPARγ activation, resulting in RTC cell death through a Sirt1-mediated PPARγ deacetylation. Therefore, the actions of PFOS through PPARγ activation or suppression might be tissue or species dependent in various experimental settings.

PFOS induced oxidative stress not only by increasing the activation of NADPH oxidase but also by eliminating the induction of antioxidative enzymes (i.e., Gpx-1, SOD-1, and catalase). We further demonstrated that PFOS reduced PPARγ binding to PPRE in the promoter region of these antioxidative enzymes, resulting in the suppression of mRNA induction. This event is triggered by Sirt1-mediated PPARγ deacetylation in PFOS-treated RTCs, which can be prevented using additional L-carnitine and Sirtinol treatments (Fig 4). Additionally, Sirt1 induction by PFOS deacetylated p53 and increased its interaction with Bax, resulting in the increase of cytosolic cytochrome C and caspase 3 activation (Fig 5). This is supported by a study showing that SIRT1 blocks nuclear translocation of p53 induced by ROS and triggers mitochondrial dependent apoptosis [33]. Additionally, the effect of PFOS on p53-mediated apoptosis can be prevented by RGZ and L-carnitine through the downregulation of Sirt1, suggesting a feedback loop of PPARγ in Sirt1 induction (Fig 5). Our results are consistent with those from a previous study, in which PPARγ interacting with Sirt1 can modulate Sirt1 transcription by binding to its promoter as a negative feedback loop in Sirt1 induction [49].

Given the importance of Sirt1 in the process of energy metabolism and cell survival, Sirt1 has been implicated in various human diseases. Notably, the results of our study showed that PFOS caused aberrant cytosolic Sirt1 accumulation in RTCs (Fig 1C). The cytosolic accumulation of Sirt1 has been observed to be a special feature in various cancer cells. Moreover, Sirt1 has been shown to be associated with epithelial mesenchymal transition although its role remains contradictory. Therefore, whether the aberrant cytosolic accumulation of Sirt1 caused by chronic PFOS exposure leads to renal fibrotic damage and renal oncogenesis warrants further investigation.

In conclusion, to our knowledge, this is the first study showing that the involvement of Sirt1 and PPARγ caused p53-mediated apoptosis by p53 deacetylation and the downregulation of antioxidative enzymes by PPARγ deacetylation and inactivation in PFOS-treated RTCs. In addition, PFOS elevated oxidative stress by inducing NFAT3 hypophosphorylation in RTCs. Of note, therapeutic intervention by using L-carnitine prevented the deleterious effect of PFOS both *in vitro* and *in vivo* through a Sirt1/PPARγ-dependent mechanism.
